# Burnout, stress and intentions to leave work in New Zealand psychiatrists; a mixed methods cross sectional study

**DOI:** 10.1186/s12888-022-03980-6

**Published:** 2022-06-06

**Authors:** Charlotte N. L. Chambers, Christopher M. A. Frampton

**Affiliations:** 1 Director, Policy and Research, Association of Salaried Medical Specialists, PO Box 10763, Wellington, 6143 New Zealand; 2 University of Otago, Christchurch, New Zealand

**Keywords:** Psychiatrists, Wellbeing, Burnout, Retention, Job satisfaction

## Abstract

**Background:**

Demand for mental health services in New Zealand and internationally is growing. Little is known about how psychiatrists are faring in this environment. This study aimed to investigate wellbeing of psychiatrists working in the public health system in New Zealand, identify the main risk factors for work-related stress, gauge perceptions of how workload has changed over time, assess job satisfaction and whether individuals intend or desire to leave their work.

**Methods:**

Psychiatrists working in New Zealand who were also members of the Association of Salaried Medical Specialists were invited to participate in an online survey. Main outcome measures were degree of burnout and stress experienced at work. Supplementary measures included perceived workplace demands and levels of support. Predictor variables included perceptions of changes to workloads over time, degree of job satisfaction and intentions to leave work. Logistic regression assessed characteristics associated with burnout and job satisfaction as well as intentions to leave work. Free text comments were analysed thematically alongside quantitative trends.

**Results:**

368/526 responded (70% response rate). 34.6% met the criteria for burnout and 35.3% scored with high work stress. There were no significant patterns of association with demographic variables but significant correlation with all but one predictor variable; having experienced a change to the demands of the on-call workload. 45% agreed they would leave their current job if able and 87% disagreed that they are working in a well-resourced mental health service. Respondents emphasised the impact of growing workloads and expressed concerns about their ability to provide optimal care in these circumstances.

**Conclusions:**

High burnout appears to affect one in three psychiatrists in New Zealand. Many attribute their feelings of burnout to demand for their services. These findings may assist with better workforce planning for psychiatry and emphasises potential consequences of demand for and poor resourcing of mental health services for the retention and wellbeing of doctors in psychiatry worldwide.

## Background

In December 2018, the New Zealand Mental Health and Addiction Inquiry released its report into the state of mental health services in New Zealand. The report stated that “… demand for specialist services [is expected to] reduce as issues are dealt with earlier, before they escalate.” [1]. Fast forward to present day, national statistics suggest demand for mental health and addiction services (MHA) has not reduced. The 2019/20 New Zealand Health Survey reported that the prevalence of very high or high psychological distress has increased to 7.4% of adults since 2011/12 [2] and another report released in 2020 reported 9% prevalence of high or very high mental distress [3]. Funding for mental health in New Zealand, however, remains set for an estimated 3% of the population requiring access to specialist mental health services [1]. In 2019, New Zealand had the lowest number of practising psychiatrists per capita when matched with 10 other comparable OECD countries [4]. New Zealand also has a high rate of reliance on international medical graduates (IMGs) to staff specialist mental health services. New Zealand medical council data suggests that in 2020, 76.9% of new psychiatrist vocational registrations were doctors from overseas [5].

These issues are neither particular to New Zealand, nor are they new. A recent study focusing on mental health services in Australia noted that “the spectres of inadequate staffing, facilities and resources lour over everyday practice and contribute to an enduring sense of futility of effort. It is often simply not possible to provide optimal psychiatric care within existing public mental health services. Problems abound at all levels” [6]. Other research has reiterated concerns with the growing demand for psychiatric care, and the challenges for trainees posed by COVID-19-related lockdowns and associated changes to training programmes [7]. While rates of recruitment appear to be relatively stable, the demand for mental health services is continuing to increase worldwide [8].

Studies on New Zealand psychiatrists throughout the mid-2000s documented concerns with levels of burnout and prefaced issues with growing patient demand, reduced administrative support and burgeoning administrative protocols [9, 10]. In 2015 psychiatrists working in the public health system were found to have the second highest rate of patient-related burnout as defined by the Copenhagen Burnout Inventory (CBI) [11] in a nationwide study [12]. In a follow-up study in 2020, New Zealand psychiatrists were found to have rates above the burnout survey average, but no significant changes to their occupational rate of burnout when compared to the 2015 study [13].

Burnout and work-related stress are two distinct but interrelated indicators of psychosocial wellbeing. While often referred to synonymously in the literature, burnout as defined by Maslach and Leitner, [14] is “a psychological syndrome emerging as a prolonged response to chronic interpersonal stressors on the job” (p103). Burnout is thus a syndrome which develops gradually as a consequence of high levels of stress at work [15] and can be understood as a “nuanced stress reaction” [16]. In 2019 the definition of burnout was changed by the World Health Organisation (WHO) to recognise burnout as an occupational syndrome with the International Classification of Diseases diagnostic manual now defining burnout as “resulting from chronic workplace stress that has not been successfully managed”. Stress is defined for the purposes of this study as referring to work-related or occupational stress, that is, an individual’s response to demands and conditions of work [18]. Understanding both levels of work-related stress and levels of burnout is anticipated to provide a good perspective on the psychosocial wellbeing of a study population with regard to their experiences of work [19].

While screening tools such as the CBI and the Maslach burnout inventory (MBI) are not clinical diagnostic tools [20, 21], surveys that signal a high prevalence of burnout in a population warrant serious consideration and attention as indicators of systemic issues. As other studies have demonstrated, there are close associations between burnout and intentions to leave work [22] working through illness [23], suicidal ideation [24, 25] and quality of patient care [26]. Burnout has significant associations with sleep deprivation and is in turn related to the likelihood of making clinically significant medical errors [27]. Research into conditions of work for mental health professionals emphasises exposure to high workloads, challenging clinical settings and trauma as likely to elevate psychiatrists’ propensity for burnout[28]. It is also possible that psychiatrists suffering from burnout may have negative feelings regarding their patients, poorer communication, and higher levels of avoidant behaviour [10, 29].

To the best of the authors’ knowledge, there have been no recent studies explicitly focused on the degree of burnout, perceived levels of occupational stress and workplace demands experienced by psychiatrists in New Zealand. There have also been no recent studies combining these quantitative indicators with qualitative data to illustrate what these indicators mean in terms of individual impact. This research seeks to fill this knowledge gap; specifically, this study aims to quantify degree of burnout, levels of occupational stress and work-demands, degree of job satisfaction as well as possible consequences in terms of intentions to leave work. It also aims to gauge perceptions of how workload has changed over time as well as describing how individuals are coping with their experiences of work.

## Methods

The study involved members of the Association of Salaried Medical Specialists (ASMS) working in psychiatry (and associated sub-specialties) at District Health Boards (DHBs). DHBs provide health care for geographically defined populations within the New Zealand public health system and are the main employers of health professionals working in the public sector. Psychiatrists are the fourth largest occupational group of the New Zealand specialist medical workforce after General Practice, Internal Medicine and Anaesthesia. According to recent New Zealand Medical Council Data, there were 671 vocationally registered psychiatrists working in New Zealand and 60% of these were international medical graduates [5]. The ASMS is the professional association and union for senior doctors and dentists in New Zealand. At the time of the survey the ASMS represented over 90% of all senior doctors and dentists and other non-vocationally registered medical and dental specialists employed within the New Zealand public health system. All 526 members of the ASMS with psychiatry and psychiatry sub-specialties specified as their medical field were asked by email to take part in an anonymous electronic survey in May 2021. 368 participants completed the survey giving a response rate of 70%. An application for ethics approval for the study was made to the national Health and Disability Ethics Committee (HDEC) convened by the New Zealand Ministry of Health. Due to the anonymous nature of the survey and the optional nature of the request to participate, the research was deemed by the committee to be outside of scope for requiring ethics approval. Participants were given an explanation in the preface to the survey that participation in the survey was proxy for informed consent and provided with an explanation as to how the resultant data would be used, including in publications.

The main outcome measures were degree of burnout and degree of stress experienced at work. Rather than incorporating a full burnout screening tool in this survey, burnout was measured using the single item measure of emotional exhaustion from the Maslach Burnout Inventory, namely the question: “I feel burned out from my work” (7-point Likert scale ranging from *Neve*r to *Daily*). High levels of burnout were defined as occurring at least once a week or more, or a score of 5 or higher on the Likert scale [30]. Previous research has established this single-item measure as a consistent and validated indicator of the degree of burnout in an occupational group [30]. Stress at work was measured using a single item question “In general, how do you find your job?” (5-point Likert scale ranging from *not at all stressful* to *extremely stressful*). Previous research has also validated this single item measure of work-related stress as providing an overall indication of job stressfulness [31]. Supplementary outcome measures of occupational characteristics which can also inflect psychosocial wellbeing were taken from the UK Health and Safety Executive Management Standards (HSE) survey. The HSE has different dimensions of demands, control, support, role, change and relationships and is designed to facilitate identification of work characteristics which contribute to psychosocial wellbeing [32]. This survey used the specific measures of perceived level of workplace demands (eg. I have to work very intensively, 5-point Likert scale ranging from 5 = never to 1 = always), and levels of peer and managerial support (eg. I am supported through emotionally demanding work and I get the help and support I need from colleagues 5-point Likert scale ranging from 1 = never to 5 = always) [33] Total scores for each of these three subscales were calculated and the scores for workplace demands reverserd so that high scores reflected fewer demands. Average HSE scores for these three dimensions were benchmarked against averages determined in a UK study [34] and a previous survey on ASMS members [35]. The study also assessed degree of job satisfaction, measured with a validated single item question (“Considering everything, how satisfied are you with your job?” 5-point Likert scale ranging from 1 = *very dissatisfied* to 5 = *very satisfied*) [36] and two pre-validated questions probing turnover intentions( “If I were able, I would leave my current job” and “I plan to leave my job within the next 6 months” 5-point Likert scale, 1 = *strongly disagree* and 5 = *strongly disagree*) [37].

To gauge perceptions of how workload and work demands had changed over time, the survey asked respondents who had been working in New Zealand when the 2018 Mental Health and Addiction Inquiry was released to recall their experiences of work at this time. A statement from the report was provided for participants, and respondents were asked to recall and compare current complexity, demand for specialist services, size of caseload and on-call demands with their workloads in 2018.

The survey also asked participants to subjectively assess whether they feel they are currently provided with enough administrative support, whether they feel their mental health service is adequately resourced and whether they feel able to provide their preferred level of patient-centred care in the time that they have available to spend with patients. These were all assessed with neutrally phrased questions, for example, ‘Do you feel you are working in a well-resourced mental health service?’ and measured according to a 5-point Likert scale (*strongly agree* to *strongly disagree*). The survey finally asked respondents how often they end up covering their colleagues’ caseloads, how often they can access the recommended levels of non-clinical time (NCT) for their service and how often they are able to see patients for follow-up appointments within clinically appropriate timeframes. These were also assessed according to a 5-point Likert scale (*strongly agree* to *strongly disagree*).

Demographic information was sought on gender (three categories), age according to ten-year increments, place of work, sub-specialty, ethnicity, and country of primary medical qualification. These demographic categories were selected to see if any patterns existed within the overall psychiatry workforce by outcome or predictor variables. Opportunities for comment were provided at the end of each block of questions and a final comments box was provided at the end of the survey for general commentary. These free text sections of the survey were treated as qualitative data and were read in conjunction with the associated quantitative questions. This process followed the broad tenets of grounded theory with the comments open coded using NVivo (version 11) into emergent themes through iterative coding with the resultant themes understood to reflect the perspectives of the research participants [38]. Comments selected for inclusion were those that best expressed the various themes. Many of the comments alluded to more than one theme simultaneously, which allowed for an exploration of how the issues of concern were related to each other as well as the complexity and tensions of participant’s views.

Raw data Likert-scale responses were coded into numeric form and basic descriptive statistics were produced. The associations between the demographic features and key variables outlined above and the two main outcome measures, degree of burnout and degree of stress experienced at work were assessed using non-parametric methods including Spearman’s rank correlation coefficients and Kruskal–Wallis tests as appropriate. The correlations amongst the three HSE scores were assessed using Spearman’s correlation coefficients. The associations between the two dichotomized outcome variables and workplace perception measures were further explored using logistic regression analyses. These analyses enabled the estimation of the likelihood of experiencing burnout (Burnout experienced at least once a week) or stress (very or extremely stressful) at different levels of the workplace variables. A two-tailed *p*-value < 0.05 was taken to indicate statistical significance and all analyses were undertaken using SPSS v27.

## Results

### Participants

Of the 526 selected ASMS members, 368 individuals responded (70% response rate) and 147 (30%) left comments for qualitative analysis at the final section of the survey. Varying numbers of respondents left comments at the end of each section of questions and *n* is specified where these comments are discussed. Analysis was undertaken on the highest number of complete responses for each question with *n* specified where relevant. The demographic profile of respondents is detailed in Table 1. Respondents were mainly international medical graduates (61%), aged in their 50s (37%), Pākehā/NZ European (44%), and commonly working in adult mental health services (37%).

**Table 1:** Sample profile characteristics

Variable	Frequency	Percentage
**Gender**	**Total *n* = 350**	
Male	174	49.7%
Female	159	45.4%
Prefer not to answer	9	2.6%
Gender diverse	8	2.3%
**Age Group**	**Total *n* = 348**	
30–39	32	9.2%
40–49	84	24.1%
50–59	129	37.1%
60–69	86	24.7%
70 and over	10	2.9%
Prefer not to answer	7	2.0%
**Ethnicity**	**Total *n* = 340**	
Päkehä/NZ European	151	44.4%
European	83	24.4%
Indian	30	8.8%
South African	24	7.1%
Other ethnic groups (NZ Maori, Chinese, other Asian, other Pasifika, other African)	52	14.9%
**Country of primary medical qualification**	**Total *n* = 341**	
New Zealand	136	39.9%
United Kingdom of Great Britain and Northern Ireland	63	18.5%
South Africa	34	10.0%
India	22	6.5%
United States of America	19	5.6%
Netherlands	9	2.6%
Germany	8	2.3%
Australia	7	2.1%
Other	43	12.6%
**Area of mental health**	**Total *n* = 347**	
Adult mental health	128	36.9%
Child and adolescent	50	14.4%
Community mental health	44	12.7%
Other	42	12.1%
Older persons/psychogeriatrics	41	11.8%
Forensic psychiatry	24	6.9%
Alcohol and drug/addiction services	18	5.2%

Analysis of the single item measure burnout scores found 34.6% with high levels of burnout using the cut-point of feeling burnt out once a week or more. Comments left in this section of the survey (*n* = 82) included reference to factors driving burnout as well as previous experiences of burnout. Others described strategies to mitigate against burnout, including taking time off work, changing their place of work, or decreasing their hours of work. The single item measure of job stress found 35.3% reporting work as either very stressful or extremely stressful. Themes identified in comments left for this section (*n* = 107) included the impact of after-hours and call work and the impact of high and growing caseloads and inadequate or mismatched resourcing. Other participants referenced the stress arising from the unpredictability of the work, fluctuating demand as well as workplace changes including decreased access to administrative support as well as fluctuating bed pressure.

### Levels of workplace demands and peer and managerial support

Table 2 outlines the mean scores for the three different variables used from the HSE management standards tool. In this presentation, low scores are negative for all three domains. The scores were highly correlated with each other (Table 3), suggesting that high demands are associated with work environments with low peer and managerial support and vice versa.

**Table 2:** Mean Health and Safety Executive scores (for all subscales low scores are negative)

Variable	*n*	Mean	SD
Perceived managerial support (5 subscale questions possible range 1–25)	365	13.57	4.31
Perceived peer support (4 subscale questions, possible range 1–20)	366	14.05	2.87
Perceived workplace demands (8 subscale questions possible range 1–40, higher scores equate to less demands)	361	20.75	5.25

**Table 3:** Correlations between HSE scores

	**Workplace Demands**	**Level of Peer Support**
Level of peer support	0.257**	-
Level of managerial support	0.224**	0.560**

^**^p < 0.001

### Intentions to leave and job satisfaction

As detailed in Table 4 almost half (*n* = 161, 45%) answered they would leave their job if they were able. Only 23% (*n* = 77) agreed or strongly agreed that they had firm plans to leave their job within the next 6–12 months. Qualitative comments in this section (*n* = 98) revealed a good deal of indecision; some noted their desire to leave the more grueling aspects of their current work, reduce hours, or find jobs that had better staffing ratios. Nearly half of all respondents reported being either satisfied or very satisfied with their job. Only 29% were either dissatisfied or very dissatisfied. Comments in this section (*n* = 82) referenced themes of passion for clinical work but anxieties from feeling overwhelmed by the demands on their services.

**Table 4:** Intentions to leave work and degree of satisfaction with job (5-point Likert scale strongly disagree to strongly agree)

Variable	Strongly disagree/disagree Frequency (%)	Neither agree nor disagree % (n)	Strongly agree/agree % (n)
I plan to leave my job within the next 6–12 months	196 (57%)	69 (20%)	77 (23%)
If I was able, I would leave my current job	131 (37%)	66 (18%)	45% (161)
	**Very dissatisfied/dissatisfied**	**Neither satisfied nor dissatisfied**	**Satisfied and very satisfied**
Considering everything, how satisfied are you with your job?	106 (29%)	95 (26%)	166 (45%)

### Time, resourcing, and workloads

**Fig. 1:**
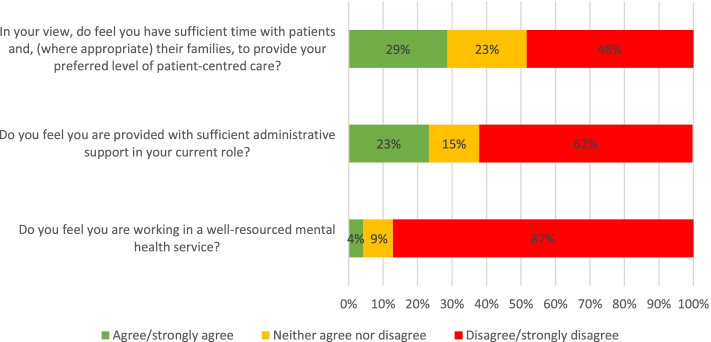
Views concerning time with patients, administrative support, and resourcing in mental health (5-point Likert scale strongly agree to strongly disagree)

As detailed in Fig. 1, while a third either agreed or strongly agreed that they had sufficient time to spend with patients to deliver patient-centred care, respondents overwhelmingly disagreed that they are provided with sufficient administrative support (62% disagree/strongly disagree) and resourcing (87% disagree/strongly disagree).

Comments in this section (*n* = 81) noted a raft of issues including poor or unreliable IT services, poor administrative support and thematic analysis emphasised a sense of general frustration with growing demands and poor resourcing. Respondents emphasised their frustration with the reduction of admin time, and statements like the following were common: “the team is down in capacity by 20% and about to lose the clinical co-ordinator but referrals and demands on service have increased. The team administrator is not able to accommodate changes. I have to use personal study time to finish admin/place clinics during peer reviews and shorten appointment times to fit more people in a week”.

Very few respondents felt able to access the recommended amount of NCT for their service. The majority agreed to some extent that they were able to see patients for their follow-up appointments within clinically appropriate time frames, while 41% of respondents felt that they always or usually covered their colleagues’ caseloads (Table 5).

**Table 5:** Additional indicators of workloads (5-point Likert scale always to never)

	**Always**	**Usually**	**Sometimes**	**Rarely**	**Never**
Frequency of covering other colleagues’ caseloads	38 (11%)	105 (30%)	166 (48%)	36 (10%)	4 (1%)
Frequency of ability to access recommended Non-Clinical Time (NCT)	10 (3%)	46 (14%)	69 (20%)	147 (43%)	80 (23%)
Frequency of seeing patients for follow up appointments within clinically appropriate time frames	12 (4%)	168 (50%)	115 (34%)	35 (11%)	4 (1%)

### Perceptions of workload change over time

**Fig. 2:**
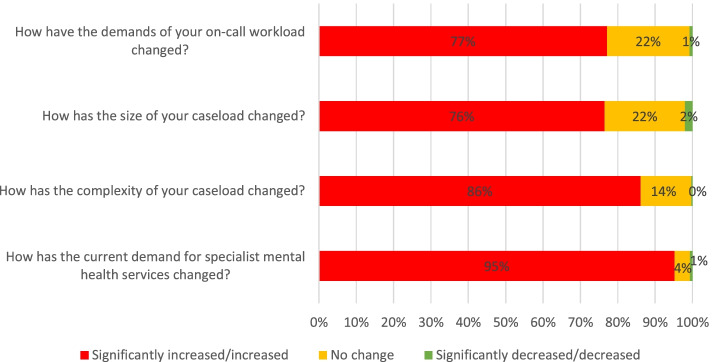
Comparison of key indicators with 2018 workloads and demands (5-point Likert scale significantly increased to significantly decreased)

Over three quarters of respondents reported an increase or significant increase to their workloads; none reported that the complexity of their caseload had decreased since 2018 (Fig. 2).

The final section of comments (*n* = 59) revealed themes focussed on the consequences of this growth in demand and concerns from clinicians for the impact on the quality of care for mental health patients. Some described feeling they had little choice but to discharge patients before they were ready, resulting in greater workload pressures down the line: “there are less and less resources available to serve the patient. There is a pressure to treat the 'acute' condition and then discharge.”; and “we often feel like patients are being discharged to the community to fail. This failure takes the form of suicide, homicide, estrangement & homelessness.”

Table 6 presents a summary of qualitative comments left throughout the survey grouped by quantitative variable.

**Table 6:** Illustrative comments for core quantitative trends and themes

Theme/variable	Illustrative comment
Burnout as a consequence of work pressure	“I frequently work overtime at the end of the day to feel that I’m on top of my work. If urgent things happen this may not be possible, and that is when I’m likely to feel burnt out. This is on top of the stress of working long hours several days in succession
Stress at work	The biggest stress is lack of beds to admit people when working on-call as well as the general risks of caring for people who at times may be violent or suicidal as part of their illness
Intentions to leave work	“I think the mental health system in New Zealand is so broken that no matter where you work, you experience the same levels of disillusionment” “If I could I’d work in mental health but not in a clinical role because of work pressure and stress”
Degree of job satisfaction	“I like seeing people and helping them. I do not like feeling that I could do better with both my patients and the service as a whole”
Time, resourcing and workloads	“My caseload is 150 + patients. Well above average for other parts of the service. This is reflective of significant population growth. I highlight these concerns regularly, but additional resource is not forthcoming. I submitted data for service sizing over two years ago, without a response yet from management.”
Growth in workload over time	“I have worked for this DHB for twenty years this year. The amount of people we see and the complexity inexorably rises, and it gets harder and harder. I still think psychiatry is a good job, but most of my senior registrars arrive at our team saying they don’t want to be psychiatrists as all the psychiatrist’s jobs look awful. It is very sad. When I look back on patient files, I am reminded how much care we could provide, 5 10 and 15 years ago to specific patients compared to now.” “If you are able to meet time frames for follow up appointments, this means that care is suboptimal”

### Correlations between variables and main outcome measures

Non-parametric Spearman’s correlations found significant associations between all variables and the degree of work-related stress experienced in the role and whether individuals scored as burnt out. A significant correlation was also found between work-related stress and burnout; 0.65, *p* < 0.001). Table 6 lists the correlation scores between the variables. The exception to this was whether people felt that the demands of their on-call workload had changed; this was not positively associated with either finding the work stressful (*p* = 0.358) or scoring as burnt out (*p* = 0.111). Negative correlations were found between the positively worded questions and the main outcomes; these are highlighted in red in Table 7. These negative associations suggest that having good support, ability to access NCT and having sufficient time with patients, low demands, and high peer support, are all correlated with lower burnout and stress scores.

**Table 7:** Non − parametric Spearman’s correlation coefficient scores between organisational variables and the two main outcome measures

	**Degree of stress associated with job**	**Scoring as burnt out from work**
If I was able, I would leave my current job	0.484^**^	0.553^**^
I plan to leave my job within the next 6–12 months	0.267^**^	0.294^**^
Considering everything, how satisfied are you with your job?	0.533^**^	0.609^**^
Do you feel you are provided with sufficient administrative support?	− 0.223^**^	− 0.208^**^
Do you feel you are working in a well-resourced mental health service?	− 0.283^**^	− 0.251^**^
In your view, do feel you have sufficient time with patients to provide your preferred level of patient − centred care?	− 0.294^**^	− 0.318^**^
How often do you end up covering other colleagues’ caseloads?	0.236^**^	0.318^**^
How often are you able to access the recommended non-clinical time for your service?	− 0.294^**^	− 0.224^**^
How often are you able to see patients for follow-up appointments within clinically appropriate timeframes?	− 0.275^**^	− 0.257^**^
How has the current demand for specialist mental health services changed?	0.176^**^	0.154^**^
How has the complexity of your caseload changed?	0.297^**^	0.232^**^
How has the size of your caseload changed?	0.205^**^	0.186^**^

^**^p < 0.01

Regression analysis results are detailed in Table 8; all factors were significantly associated; having time to provide desired levels of patient-centred care and being able to see patients within the clinically recommended time frame for follow-up appointments had the biggest impact on the likelihood of reducing burnout; being able to accomplish these two factors reduced the likelihood of experiencing burnout by nearly 46% and 48% respectively.

**Table 8:** Regression analysis results displaying odds ratios by variables and main outcomes

	**Degree of stress associated with job (Very or Extremely stressful)**				**Frequency of feeling burnt out (≥ 1 time per week)**			
	***p*-value**	Odds ratio*	**Lower 95%**	**Upper 95%**	***p*-value**	Odds ratio*	**Lower 95%**	**Upper 95%**
**Desire to leave job**	< 0.001	2.274	1.820	2.840	0 < 0.001	3.213	2.454	4.205
**Plan to leave job**	< 0.001	1.526	1.274	1.829	0 < 0.001	1.709	1.417	2.061
**Work in well-resourced MHS**	< 0.001	0.585	0.429	0.798	0 < 0.001	0.489	0.350	0.682
**Degree of job satisfaction (Negatively worded variable)**	< 0.001	3.402	2.564	4.514	0 < 0.001	5.029	3.612	7.000
**Has admin support**	0.001	0.719	0.590	0.878	0 < 0.001	0.635	0.516	0.782
**Has sufficient time with patients**	< 0.001	0.652	0.525	0.810	0 < 0.001	0.543	0.432	0.682
**Covering colleagues’ caseloads**	0.001	1.602	1.224	2.097	0 < 0.001	2.114	1.587	2.815
**Ability to access NCT**	< 0.001	0.626	0.495	0.790	0 < 0.001	0.637	0.505	0.803
**See patients for follow-up within clinically appropriate timeframes**	< 0.001	0.545	0.403	0.736	0 < 0.001	0.517	0.382	0.702

Analysis found no significant associations with demographic variables and any of the two main outcome measures, with the exception that New Zealand trained graduates perceived their workplace demands to be higher than those who were IMGs. The Kruskal–Wallis summed rank test did not reveal significant differences in either burnout or degree of stress related to the job by place of work.

## Discussion

This study presents an in-depth account of the psychosocial wellbeing of psychiatrists working at the frontline of New Zealand’s mental health service. It extends previous research into the wellbeing of psychiatrists in New Zealand and presents data of international relevance. The 70% response rate suggests a high level of motivation amongst psychiatrists working in New Zealand to share their experiences of working in the public system.

The burnout scores in this study are slightly higher than those in other research on psychiatrists; a similar study into rates of burnout in psychiatrists working in Ireland found 33% suffering from burnout [39]. Kumar’s 2007 study into New Zealand psychiatrists also reported 33% as suffering from high emotional exhaustion [40] and reported 35% burnout in their 2012 research [41]. The single item measure of burnout used in this survey is not as fulsome a measure as the full MBI or CBI, but analysed with the degree of stress experienced at work, it provides good overall insight into psychiatrist wellbeing.

This study adds to the literature demonstrating the pervasiveness of burnout as an entrenched feature of the senior medical workforce and a pressing issue of concern for those working in psychiatry. The consequences of burnout in terms of risks of medical error and possible consequences for the quality of patient care has been well substantiated in other studies [42, 43]. Research into New Zealand SMOs has postulated a negative relationship between degree of burnout and degree of patient-related empathy [44]. While the cross-sectional nature of this research means a causal relationship cannot be inferred, the possibility that burnout and stress may have an impact on the quality of patient care must be taken seriously by those involved with staffing and resourcing of services.

The widespread experiences of burnout and work-related stress suggested that psychiatrists across the board are equally at risk of stress and burnout. Correlation analysis with non-demographic variables found that experiencing burnout and work-related stress was significantly correlated with all predictor variables except for having experienced a change to the demands of the on-call workload (*p* = 0.358 and 0.111 for stress and burnout respectively). This suggests clinician’s wellbeing is significantly associated with perceptions of demand, how well resourced their mental health service is felt to be, and whether they feel they are provided with support. All these factors are recognised to constitute significant sources of stress and are understood as contributing factors in burnout research more widely [45–47]. The findings of this research are consistent with other studies where staff shortages, inadequate resourcing and a disjunction between the views and priorities of managers are emphasised as contributing to burnout [45, 48].

The odds ratio analysis enabled a finer grained exploration as to key risk factors as well as potentially protective factors associated with work-related stress and burnout. Feeling that clinicians had adequate time with patients and being able to see patients for follow up appointments within clinically recommended guidelines appear protective against stress and burnout. These factors tally with the qualitative comments left which emphasise the desire for clinicians to provide the best quality care for their patients while simultaneously finding this challenging to do because of heavy workloads and varying constraints upon their time. These connections between wellbeing and conditions of work are substantiated in other studies where factors such as workload, degree of control, recognition or reward for work effort, and having a sense of community and support in the workplace all shape propensity for burnout [14]. The strong associations between indicators of resourcing and demand and ability to provide quality patient care emphasises the significance of well-resourced mental health services for the wellbeing of those tasked with providing care. Other noteworthy factors include the positive impacts on wellbeing of enabling clinicians to access their NCT. One study found that regular access to as little as 2 h a week of NCT is likely to reduce the likelihood of doctors experiencing burnout [49].

Another aspect that the odds-ratio analysis highlighted was the importance of having adequate administrative support. Those respondents who reported good levels of administrative support were 28% less likely to find their job stressful and 37% less likely to experience burnout. This emphasis on administration is consistent with other studies in the New Zealand context such as that by Fischer and Kumar who note that lack of administrative support can also include the notion of “an aggressive administrative environment” [9 p420]. Again, while causality cannot be inferred, these findings suggest that readily achievable and relatively cost-neutral changes such as ensuring access to NCT and provision of more comprehensive administrative support would likely pay dividends in terms of improving wellbeing for this critical workforce.

The inclusion of the HSE management standards tool in this study provides further insight as to the connections between wellbeing and conditions of work. The correlation analysis suggests that emotional exhaustion and job stress are strongly associated with working situations where work demands are high, workloads are growing, and support levels are low. Comparison of mean HSE scores with a UK study and a previous ASMS survey finds that levels of workplace demands are higher in this current research while levels of support, both peer and managerial, are lower. The low scores for receiving supportive feedback and being supported through emotionally demanding work provide indicators as to what factors can be improved in workplaces. In line with other research, working in environments where clinicians feel supported by managers and have strong sense of collegiality are protective factors for wellbeing and have the capacity to ameliorate feelings of stress [35, 50]. In contrast, many respondents in this study expressed frustration with perceived disconnect with non-clinical managers regarding the pressures and stresses faced by clinicians at the coal face.

The third objective of this research was to gauge perceptions of how workload (quantity and quality) had changed since 2018. The perception of the psychiatrists was overwhelmingly negative with nearly all reporting increases to the demands of their on-call workloads, caseloads, and the complexity of patients seen. Many responded to the question with comments that expressed incredulity at the predictions in the 2018 mental health inquiry. For example, one psychiatrist noted “I despair when I read that statement in the report—bears no resemblance AT ALL to reality” (emphasis in original). The long recall period and the subjective nature of this question is a limitation of this study; further longitudinal research would be helpful to track indicators of demand over time.

Despite the moderate levels of burnout and work-related stress reported in this study, over 40% of participants stated they were satisfied with their job, with 4.6% very satisfied. This finding was consistent with that reported by Kumar et al. [41] where job satisfaction appeared to persist at a rate higher than levels of burnout. Nevertheless, as outlined in the logistic regression results, a decrease in job satisfaction was associated with a 3.4 times greater likelihood of finding the job stressful and 5.0 times greater likelihood of experiencing burnout.

The combination of the quantitative and qualitative analysis suggests most psychiatrists surveyed gain pleasure and enjoyment from their interactions with patients. Despite this positivity, however, the thematic analysis emphasised impacts on their job satisfaction from what was described as a system set up to fail patients. One of the potential consequences of low job satisfaction was manifest in the respondents’ views concerning desire and plans to leave their work. There is an acknowledged relationship between low job satisfaction and intentions to leave medicine [51, 52] as well as between intentions to leave medicine and rates of stress and burnout in doctors [47]. While understanding the relationship between burnout and work stress and intentions to leave psychiatry was not a specific focus of the research, the association found between low job satisfaction and thoughts of and intentions to leave work is noteworthy. This association is consistent with established literature and reinforces what is potentially at stake for a mental health workforce already experiencing staffing shortages and difficulties with growing demand for specialist interventions.

The application of existing, pre-validated measures such as the single item measure of burnout, degree of job stress and the use of the HSE indicators of workload, peer and managerial support ensures comparability of these results with other peer-reviewed literature and an objective assessment of the pressures this psychiatry workforce is under. The inclusion of questions pertaining to intentions to leave, ability to access admin support and non-clinical time, as well as subjective perceptions of changes to workload over time add depth to the likely drivers of the psychosocial wellbeing of this workforce. Application of the odds ratio analysis enables insight as to what factors may have the greatest impact in terms of instituting wider change. The themes identified in the comments provides insight as to the consequences of the quantitative trends for clinicians and patients alike. Further in-depth qualitative studies using either individual interviews or focus groups would be extremely helpful to probe these issues further.

## Conclusions

This study provides new information on the relationship between key indicators of wellbeing, levels of job demand, perceptions of workload over time and job satisfaction in the cornerstone of New Zealand’s mental health workforce. The findings of this research, that over a third of psychiatrists in this study are experiencing high levels of burnout and work-related stress, and many are struggling with high work demands and moderate levels of peer and managerial support – are concerning. All of these factors are known to be associated with increased intentions to leave work and for those suffering from burnout, recovery is known to be a lengthy process.

The near consensus of respondents regarding the growth in workloads, on-call demands, and patient complexity is also of concern and must serve as a clarion call to those with responsibility for workforce planning. Ensuring that psychiatrists have clearly defined workloads which accounts for the growth in demand and patient acuity, is fundamental to achieving safe work, psychiatrist wellbeing and job satisfaction. This research provides pointers as to what factors could be readily adjusted to improve working conditions. The provision of better administrative support, functional and connected IT systems are all highlighted in this research and would bring significant benefits to improving wellbeing.

The persistence of relatively high job satisfaction for psychiatrists involved in this research is heartening. Psychiatrists continue to report significant satisfaction from their ability to provide high quality care for those in need. Nevertheless, the findings of this research that just over half of those surveyed are always or usually able to see patients for follow-ups within clinically appropriate timeframes and that many feel discharges are made too soon due to sheer volume of demand is of concern. Psychiatrists do not wish to discharge their patients to fail. This research makes clear the emotional toll for clinicians as well as the serious possible consequences for patients.

Finally, this study is of key relevance for those tasked with workforce planning for psychiatry and emphasises potential consequences of the high demand for mental health services for the retention and recruitment of doctors in psychiatry. The high response rate to the survey and the use of pre-validated instruments allows for comparison with other studies as well as ensuring rigour and confidence in these findings. Nevertheless, the cross-sectional design of the survey and inclusion of subjective self-report measures with a relatively long recall period are limitations of this research. This also means that the associations noted between core variables cannot be causally determined.

Since the completion of this study, COVID-19 continues to have a significant impact on the wellbeing of the health workforce in New Zealand. It is likely that demand for mental health services will continue to escalate as well as the pressures on health care workers to continue to provide care in these uncertain and stressful times. This means that some of the pressures outlined in this research are likely to worsen in the short term.

## Data Availability

The datasets generated and/or analyzed during the current study are not publicly available due to the commitments made to research participants but may be made available from the corresponding author on reasonable request.

## Abbreviations

ASMS: Association of Salaried Medical Specialists
DHBs: District Health Boards
HSE: Health and Safety Executive
IMG: International Medical Graduate
IT: Information Technology
MBI: Maslach Burnout Inventory
MHA: Mental Health and Addiction services
NCT: Non Clinical Time
SMOs: Senior Medical Officers
